# Involved-field irradiation in definitive chemoradiotherapy for locally advanced esophageal squamous cell carcinoma

**DOI:** 10.1186/1748-717X-9-64

**Published:** 2014-02-26

**Authors:** Xiaoli Zhang, Minghuan Li, Xue Meng, Li Kong, Yan Zhang, Guangsheng Wei, Xiqin Zhang, Fang Shi, Man Hu, Guoli Zhang, Jinming Yu

**Affiliations:** 1 Departments of Radiation Oncology, Shandong Cancer Hospital and Institute, Shandong Academy of Medical Sciences, Jinan, China

**Keywords:** Esophageal squamous cell carcinoma, Irradiation, Nodal metastasis, Patterns of failure, Prognosis

## Abstract

**Background:**

Since there is high local failure and poor survival for unresectable esophageal squamous cell carcinoma (ESCC), the necessity of elective node irradiation is controversial. The purpose of this study was to investigate the failure patterns and survival in patients with locally advanced ESCC receiving involved-field irradiation (IFI).

**Methods:**

A retrospective study was preformed on the clinical records of patients with locally advanced ESCC, who have received IFI with concurrent chemotherapy between January 2003 and January 2009. Comparing the target volume and first sites of failure, patterns of failure were defined as in-field, out-of-field regional lymph node and distant failure. The survivals were analyzed by different patterns of failure.

**Results:**

Eighty patients were included in our study. With a median follow-up of 52.6 months, failures were observed in 76 patients. In-field recurrence, distant metastasis, and out-of-field regional failure were seen in 53.75%, 41.25%, 30% patients, respectively. There were significant differences in OS for patients with and without in-field (median OS 14.2 vs.17.4 m, P = 0.01)or distant failure(13.2 vs.15.9 m, P ≤ 0.0001), but not for out-of-field regional lymph node failure(both 14.5 m, P = 0.665).

**Conclusions:**

The solitary regional nodal failure of out-of-field was acceptable in advanced ESCC patients treated with IFI. In-field and distant failures remained the predominant patterns and negatively impacted survival more significantly. Further investigation is needed to establish the optimal radiotherapy field for these patients at advanced stage.

## Background

Esophageal cancer (EC) is the fifth most common cancer and the forth leading cause of cancer deaths in China. Different from the western countries, squamous-cell carcinoma accounts for 95% of all Chinese EC patients [1]. EC is notorious for its lymph node (LN) metastases, which LN involvement is an early process and also skip metastases are common. Nodal spread of esophageal tumors may be extensive at initial clinical presentation. More than 50% EC cases are diagnosed at locally advanced stage with obvious enlargement node, long lesion and/or serious esophageal invasion. For these patients, the results of a phase III randomized trial (Radiation Therapy Oncology Group [RTOG] 85–01) comparing chemoradiotherapy (CRT) with radiation alone have made definitive CRT to one standard treatment option [2]. But in practice, the radiation field of EC has reached no global consensus till now. In RTOG 85–01, the range of clinical target volume (CTV) was from the supraclavicular region to the gastroesophageal junction. But in RTOG 94–05, 5-cm proximal and distal margins and a 2-cm lateral margin from the borders of the gross tumor volume (GTV) were recommended [3]. The supraclavicular nodes were included only when the tumor was located in the cervical esophageal area. Some recent studies showed that the involved-field irradiation (IFI, nodal target volume included only the malignant nodes) may be feasible for some sub-group patients with esophageal squamous-cell carcinoma (ESCC). Kawaguchi found that IFI did not result in significant incidence of regional LN failure in clinical stage I thoracic EC patients [4]. For patients of EC receiving definitive radiotherapy, some researchers employed three-dimensional conformal radiotherapy (3D-CRT) without intentional elective node irradiation (ENI) and the rate of isolate out-of-field nodal failure was only 2-8% [4-6]. Moreover, some published reports indicated that serious toxicities would occur in at least 50% of patients with EC receiving concurrent chemoradiation therapy (CCRT) if ENI was adopted [3,7]. Thus, IFI may result in reduced incidence of treatment toxicities which enable more patients to tolerate the CCRT.

To date, there have been few reports on the exclusive use of IFI for locally advanced ESCC patients. The present study sought to retrospectively document the failure patterns and survival, and evaluate the feasibility of IFI for this specific population.

## Materials and methods

### Patients

From 2003, the therapeutic regimen of IFI with concurrent chemotherapy was performed for EC patients in our department. This retrospective study was preformed on the clinical records of patients with locally advanced ESCC, who have received IFI with concurrent chemotherapy of cisplatin (CDDP) and 5-fluorouraci (5-FU) between January 2003 and January 2009. Patients were excluded if they had any other malignant tumor history. The disease had been confirmed by biopsy or brushing and had not yet been treated before. Every patient had been assessed and staged by examinations including esophagogram, endoscopy, and computed tomography (CT), and some by positron emission tomography (PET)/-CT fusion scans. All of the reviewed cases had one of the following characteristics: cervical lesion, upper thoracic lesion with length more than 6 cm, T4 disease, or obvious (bulky or multiple) involvement of regional lymph nodes. The institutional review board of Shandong Cancer Hospital approved our study.

### Chemoradiotherapy

All patients had been treated with definitive chemotherapy with concurrent radiotherapy.

All radiation treatments were to be delivered as 3D-CRT or Intensity Modulated Radiation Therapy (IMRT) with standard fractionation (2.0 Gy fraction^-1^, 5 days per week); treatment plans were generated with three-dimensional planning system (ADAC-Pinnacle 3, version 5.0). The GTV was contoured on the planning CT scans by the attending radiation oncologists using all available resources, including data from esophagogram, endoscopic ultrasonography (EUS) images, diagnostic CT images and PET/CT fusion scans. The GTV was defined as any visible esophageal lesion (GTVe) and clinical involved node (GTVn). The primary criterion for node metastases was the size. The lymph nodes over 1.0 cm in the short axis or 1.5 cm in the long axis on CT scans or with a high SUVmax of FDG avid on PET images were considered to be metastatic. Other criteria, including the nodal enhancement pattern and the presence of extra-nodal tumor extension, also had been used to help assess the metastatic status. The CTVe was defined as the GTVe plus a 3.0-4.0 cm margin superior and inferior to the primary tumor and a 0.8-1.0 cm radial margin. CTVn was defined as the GTVn plus a 0.5-1.0 cm radial margin. The PTV was defined as the CTV plus a 0.5-1.0 cm margin. All organs at risk were outlined. Although, according to the RTOG 85–01, chemotherapy could help to some degree, it did not change the fact that this dose (50.4 Gy in 28 fractions) was inadequate to achieve high probability for local control. So in our study, all patients were treated with a total dose of 50- 64Gy, given in 25–32 fractions, with the median dose of 60Gy.

Chemotherapy began on day 1, concurrent with the beginning of radiation. The chemotherapeutic regimens consisted of two cycles of CDDP (75 mg/m^2^/day on Day 1) and 5-FU (700 mg/m^2^/day as a continuous infusion from Day 1 to Day 4) every21 days in all patients. Additional 1–2 (median 2) cycles of chemotherapy with the same regimens were administered for 57 patients.

### Result assessment and follow-up

The tumor response and recurrence were evaluated and classified according to the Response Evaluation Criteria in Solid Tumors (RECIST) system [8] and the final results were recorded by the follow-up data. In brief, the responses were classified as follows: complete response (CR), partial response (PR), progressive disease (PD), stable disease (SD). The overall response (RR) rate was defined as the CR rate plus the PR rate. We assessed failure models on post treatment esophagogram, endoscopy, CT, or PET/CT scans and compared those data with the original CT- based radiation treatment plans. However, when an esophageal recurrence was suspected, it was confirmed by histologic or cytologic testing. LN recurrences were diagnosed on the basis of the following findings: (1) nodes that re-appeared after complete disappearance; (2) nodes that enlarged after remaining stable; and (3) new nodes that appeared in the mediastinal or abdominal regions where no enlarged nodes had existed before irradiation. Suspected supraclavicular node recurrences were confirmed by fine needle aspiration biopsy. Patterns of failure were defined according to the first sites of failure and included in-field, out-of-field regional LN and distant failure, respectively. In-field recurrence included primary lesion and involved regional LN failure. Out-of-field regional LN failure was defined as the failure of initially uninvolved LN within the regional LN. Regional lymph nodes were defined as periesophageal LN extending from the cervical nodes to the celiac nodes [9]. The LN metastases outside the regional level were considered as distant failures. Overall survival (OS) and progression-free survival (PFS) were calculated from the first day of irradiation.

### Statistical analysis

Continuous variables were summarized by descriptive statistics such as means, standard deviations, medians, and ranges. Categorical variables were tabulated by frequency and percentage. The Kaplan-Meier method and log rank test were applied to estimate survival probabilities and compare survival, respectively. Cox proportional hazards models were fit to evaluate potential associations between OS and clinical factors. The backward selection procedure was used for model selections. SPSS version 17.0 (SPSS Inc., Chicago, IL, USA) statistical software was used for statistical data analysis.

## Results

### Patients characteristic

Complete data were available for 80 patients treated from January 2003 to January 2009. Among of them, 11 patients underwent staging of PET. Clinical and treatment characteristics were listed in Table 1.

**Table 1:** Patient characteristics

**Patient characteristics**	**N (%)**
Sex	
Male	52(65.00)
Female	28(35.00)
Age, y, median (range)	63(42–74)
Tumor category	
T1-3	63(78.75)
T4	17(21.25)
Lymph node category	
N0	21(26.25)
N+	59(73.75)
Location	
Cervical	2(2.50)
Upper thoracic	19(23.75)
Mid-thoracic	49(61.25)
Lower thoracic	10(12.50)
Tumor length, cm,	5.00(2–13)
Radiation dose	
<60 Gy	11(13.75)
≥60 Gy	69(86.25)
Median (range), Gy	60(50-64)
Adjuvant chemotherapy	
Yes	57(71.25)
No	23(28.75)
Median (range)	2(0–4)
Salvage treatment	65(81.25)
Chemotheray	32(40.00)
Palliative radiation	35(43.75)
Surgery	8(10.00)

### Patterns of failure

**Figure 1:**
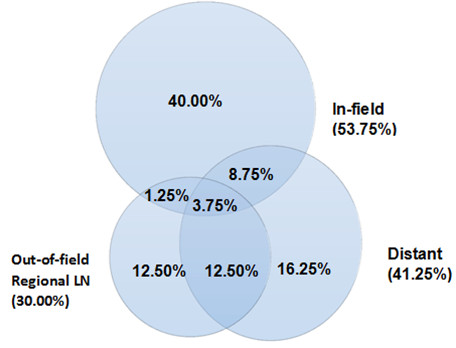
**The rate of different failure patterns.** Patterns of failures are shown based on the original radiationtreatment volumes, 53.75% within the in-field volume, 30.00% within the out-of-field regional LN, and 43.90% in the distant.

At the time of last follow-up contact in December 2011, failures were observed in 95% patients, with 53.75% of in-field, 30% of out-of-field regional LN and 41.25% of distant metastases, respectively. Among of them, twenty-one patients were observed to fail in more than one site. The sites of first failure were overlapped in some patients, which were shown in Figure 1. According to our data, 60.6% patients in distant failure group were companied with regional or in-field failure, while 58.3% cases in regional recurrence group were companied with other failures. But the in-field as only site of failure was seen in 74.4% cases among the in-field pattern group. The rate of in-field failure alone, distant failure alone, out-of-field regional LN failure alone were 40%, 16.25% and 12.5%,respectively. The most common sites of out-of-field regional LN localized in upper mediastinal (12 cases), celiac (9 cases) and supraclavicular levels (7 cases). The most common sites of distant metastases were distant LN in 17 cases, bone 6, lung 5 and liver 5, respectively. The details of failure patterns were listed in Table 2.

**Table 2:** The failure patterns

**Failure patterns**	**Patients (n)**
In-field with or without others	43
In-field alone	32
Out-of-field with or without others	24
Out-of-field alone	10
Supraclavicular	3
Mediastinal	3
Celiac	4
Distant with or without others	33
Distant alone	13

### Response and survival

**Figure 2:**
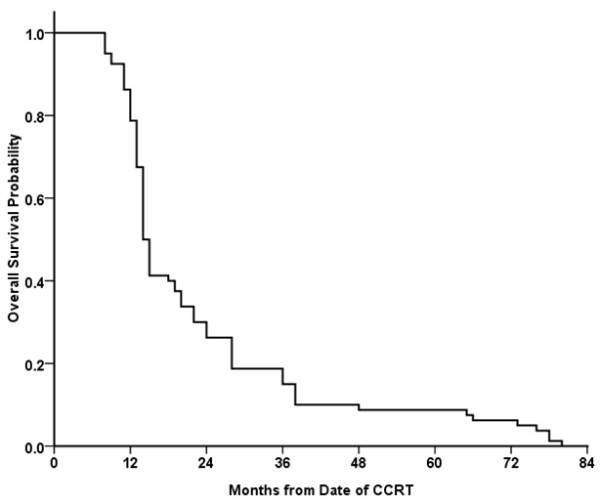
**The overall survival data for all patients in this study.** The 1-year, 2-year, 3-year OS rates were 86.3%, 30.0%, 18.8%, respectively.

**Figure 3:**
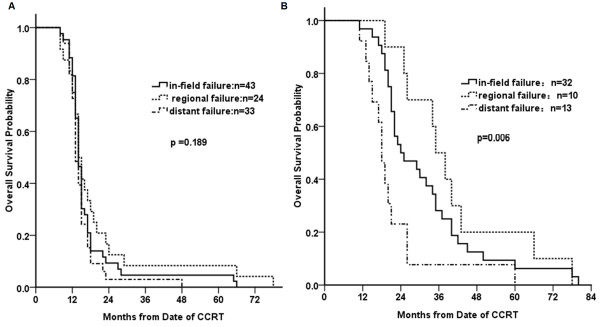
**The overall survival data for patients with different failure patterns (A) and solitary failure patterns (B).** Kaplan-Meier estimates of OS are shown according to different failure patterns **(A)** and solitary failure patterns **(B)**. No significant difference in OS was found for patients with different failure patterns (*P* = 0.189). But there was a significant difference in OS for patients with solitary failure patterns (*P* = 0.006).

The RR rate was 85%, with CR in 23.75% of patients, and PR in 61.25% of patients. At a median follow-up period of 52.6 months (95% confidence interval [CI], 46.1-56.7 months), the median PFS time was 11.3 months (95% CI, 8.8-13.2 months) with 1-year, 2-year, 3-year PFS rates of 41.3%, 18.9%, 11.3% in these advanced stage patients, respectively. And the median OS time was 14.4 months (95% CI, 13.4-15.9 months). The 1-year, 2-year, 3-year OS rates were 86.3%, 30.0%, 18.8%, respectively (Figure 2). Univariate analysis showed that OS was associated with baseline T status (P = 0.027) and N status (P = 0.016). According to different failure patterns, the median survival times were 14.2 months for in-field, 14.5 months for out-of-field and 13.2 months for distant failure group, respectively. No significant differences were found (P = 0.189) among these groups (Figure 3A). The OS time of in-field, regional and distant failure alone were 24.5 m, 35.3 m and 18.2 m (P = 0.006, Figure 3B), respectively.

**Figure 4:**
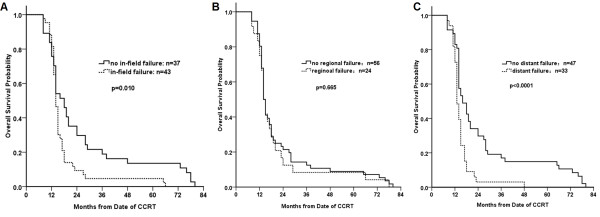
**The overall survival data for patients with and without specific failure pattern.** Kaplan-Meier estimates of OS are shown according to specific failure pattern. Significant difference in OS was found for patients with and without in-field (*P* = 0.010, **A**), and distant failure (*P* < 0.0001, **B**), but not in out-of-field regional failure (*P* = 0.665, **C**).

To observe the contribution of different failure patterns to survival, we compared the survival of patients with specific pattern to all the other patients. The median OS time for patients with in-field failure was 14.2 months (95% CI, 13.2-14.9) versus 17.4 months for those with non in-field failure (95% CI, 14.8-19.4; P = 0.01, Figure 4A). Having a distant failure also influenced OS time in our study. Patients without distant failure achieved a better OS than those with distant failure (15.9, 95% CI, 12.4-16.2 vs.13.2, 11.9-15.2 months; P < 0.0001, Figure 4C). But no significant difference was found in the median OS time for patients with or without out-of-field regional LN failure pattern (95% CI, 14.5, 13.2-16.2 vs. 14.5, 12.4-15.7 months; P = 0.665, Figure 4B). For the 10 cases of solitary regional nodal recurrence, the median PFS and OS time were 13.2 (range 4–38) and 35.6 (range 11–45) months, respectively.

### Salvage therapy

Sixty-five patients have undergone salvage treatment after treatment failure,which including chemotherapy, palliative radiotherapy and surgery. All of the 10 cases with solitary regional LN failure received salvage treatment. Among of them, 2 cases with supraclavicular recurrence received lymphadenectomy and chemotherapy, 4 cases (1of supraclavicular, 3 of upper mediastinal) with radiotherapy only, 4 of celiac failure with chemotherapy and radiation. After the salvage treatment, 4 of the 10 cases experienced more than 24 months survival period.

## Discussion

In this study, we investigated treatment results and patterns of first site failure after IFI for locally advanced ESCC. We found that in-field failure and distant metastasis remained the predominant failure patterns in these cases. Among the 76 patients with failure, the rate of out-of-field regional LN failure alone was 12.5%. Zhao et al. have evaluated the results of IFI for EC patients with 3D-CRT. The rate of in-field recurrence was 44%, but only 8% cases were isolated out-of-field nodal recurrences [6]. Uno et al. reported that a radiotherapy PTV including only clinically-involved lesion for patients with EC aged 75 and older, resulted in no isolated LN recurrence [10]. The concern was that the persistence of disease was the greatest cause of treatment failure (despite therapy), and 26% of patients receiving combined therapy experienced local failure. Published data has reported that regional LN failure rates ranged from 5% to 15% for ENI in EC patients receiving radiotherapy [11]. In conclusion, for ENI or IFI,the regional LN failure was not the main pattern of recurrence in these advanced stage ESCC patients.

In present study, we also found a longer median survival time in patients with regional failure, but there was no statistic difference in OS time between different failure patterns. It was mainly caused by the overlap of failure sites in this population. According to our data, more than half (58.3%) cases were companied with other sites of failure in regional recurrence group. When we analyzed the survival with solitary failure pattern, a longest median survival time was found in the group of regional failure alone. Our further analysis showed that the patients with in-field or distant failure had worse survival. But the OS for patients with and without out-field failure showed no statistical significant difference (P = 0.665). These data may indicate that, compared with regional failure, the in-field and distant failure negatively impacted survival more significantly. The relatively high incidence of in-field recurrence and distant metastasis may mask regional nodal failures because many of the patients have died before their regional nodal failure became clinically apparent or threat to life. So the relatively high regional control acquired with ENI may not translate into the benefit of OS, especially for advanced stage cases.

And on the other hand, if only the solitary regional nodal failure occurred, a salvage treatment may provide better survival. In our study, 40% (4/10) of the cases with solitary regional failure experienced more than 24 months survival period after the salvage treatment. Another two studies also supported the efficacy of the salvage treatment for solitary regional node recurrence. Yano et al. reported the result of treatment for 35 patients with the cervical LN recurrence, and concluded that substantial survival could be attained by salvage local-regional treatment if it was a solitary node recurrence [12]. Watanabe et al. performed salvage cervical lymphadenectomy followed by adjuvant chemotherapy for all five patients with recurrence limited within the cervical nodes, and observed no disease relapse in a median follow-up period of 54 months. The median survival period after the salvage treatment was 60 months [13]. So the salvage treatment after regional LN failure alone may mitigate its adverse effect on survival.

Although all the patients included in our study were in locally advanced stage, we could still see a few patients with long-term survival, which indicated a good response to CCRT. Previous studies have demonstrated that only patients with histological complete response can acquire survival benefits [14,15]. Unfortunately, most EC patients were resistant to chemoradiation, with only 20%–40% pathologic CR rate after definitive CCRT in advanced stage patients [16]. As high as 11–26% of those patients did not exhibit any morphological response, leading to a dismal prognosis with a median survival of only 9 months [17]. In our opinion, for those patients of complete responder, ENI may be appropriate to eliminate micrometastases in regional LN, which might get longer PFS. And for those non-responders or non complete responders,the ENI seems unnecessary,if the primary lesions could not be well controlled. So additional evaluations may be warranted to assess the sensitivity of patients to CCRT and then individualize treatments, thereby sparing patients unnecessary toxicity from ineffective therapy.

However, even though the in-field failure was the most common site (53.75%)of initial failure, patients in our study also experienced high rate (41.25%)of systemic failure. Thus, efforts to increase local control may not necessarily translate into improved survival unless systemic therapies also improved. The intensification of chemotherapy through the addition of induction chemotherapy had been in trial to strengthen the systemic therapy, but no consensus response was obtained until now [18,19]. To improve both local and distant control in patients with EC, new regimens must be developed. Perhaps only when the high local failure rate is addressed, the value of this approach can get fully realized.

In the present study, the failure rate of more than 90% in the whole patient group and the survival data (18.8% for 3y) appeared worse than preceding reports. Some factors may have contributed, in part, to the high failure rate and worse overall survival in our study. First, all of the patients included in our study were in locally advanced stage with wide local tumor extension and/or clinically obviously node metastases, which was the most important cause for bad prognosis. Second, EUS, PET scans and other functional images were not available for all of the cases in the trial, which might lead to diagnostic underestimation, have impacted the target volume to IFI. The microscopic disease in normal sized nodes and LN enlargement caused by benign conditions, limit the diagnostic accuracy of CT for nodal enlargement caused by EC. According to a meta-analysis, the sensitivity and specificity of CT for regional lymph node metastases were 0.50 and 0.83 in thoracic tumor, respectively [20]. In the future study, if the IFI was used, the more accurate diagnostic technique should be performed to avoid the missing of involved node. Third, ENI should be really effective for providing regional control of LN micrometastases and leading to a longer PFS, especially for responding cases who had a relatively long survival.

In summary, our study found that in-field and distant failure remained the major failure patterns in patients with locally advanced ESCC treated with IFI and impacted the survival time more significantly, while the solitary nodal failure of out-of-field was acceptable and the salvage treatment for solitary regional failure may improve outcome. The omission of elective nodal irradiation did not sacrifice OS, to some extend, which suggested the feasibility of IFI for the locally advanced cases. However, this was a retrospective study with a relatively small sample size, which may limit the generalizability of our findings. Further observations with large-scale, multi-center, prospective, stratification, randomized clinical trials are needed to verify the feasibility of IFI in this sub-group population.

## Competing interests

The authors declare that they have no competing interests.

## Authors’ contributions

XZ and ML drafted the manuscript. XM, LK, YZ, GW, XZ, MH, FS, GZ participated in data collection, and helped to analyze the data. JY participated in the coordination of the study. All authors read and approved the final manuscript.

## References

[B1] Chen W He Y Zheng R Zhang S Zeng H Zou X He J Esophageal cancer incidence and mortality in China, 2009 J Thorac Dis 2013 5 19 26 23372946 10.3978/j.issn.2072-1439.2013.01.04 PMC3547988

[B2] Cooper JS Guo MD Herskovic A Macdonald JS Martenson JA Jr Al-Sarraf M Byhardt R Russell AH Beitler JJ Spencer S Asbell SO Graham MV Leichman LL Chemoradiotherapy of locally advanced esophageal cancer: long-term follow-up of a prospective randomized trial (RTOG 85–01). Radiation Therapy Oncology Group JAMA 1999 281 1623 1627 10235156 10.1001/jama.281.17.1623

[B3] Minsky BD Pajak TF Ginsberg RJ Pisansky TM Martenson J Komaki R Okawara G Rosenthal SA Kelsen DP INT 0123 (Radiation Therapy Oncology Group 94–05) phase III trial of combined-modality therapy for esophageal cancer: high-dose versus standard-dose radiation therapy J Clin Oncol 2002 20 1167 1174 10.1200/JCO.20.5.1167 11870157

[B4] Kawaguchi Y Nishiyama K Miyagi K Suzuki O Ito Y Nakamura S Patterns of failure associated with involved field radiotherapy in patients with clinical stage I thoracic esophageal cancer Jpn J Clin Oncol 2011 41 1007 1012 10.1093/jjco/hyr069 21665908

[B5] Welsh J Settle SH Amini A Xiao L Suzuki A Hayashi Y Hofstetter W Komaki R Liao Z Ajani JA Failure patterns in patients with esophageal cancer treated with definitive chemoradiation Cancer 2012 118 2632 2640 10.1002/cncr.26586 22565611 PMC3747650

[B6] Zhao KL Ma JB Liu G Wu KL Shi XH Jiang GL Three-dimensional conformal radiation therapy for esophageal squamous cell carcinoma: is elective nodal irradiation necessary? Int J Radiat Oncol Biol Phys 2010 76 446 451 10.1016/j.ijrobp.2009.02.078 20004527

[B7] Ishikura S Nihei K Ohtsu A Boku N Hironaka S Mera K Muto M Ogino T Yoshida S Long-term toxicity after definitive chemoradiotherapy for squamous cell carcinoma of the thoracic esophagus J Clin Oncol 2003 21 2697 2702 10.1200/JCO.2003.03.055 12860946

[B8] Therasse P Arbuck SG Eisenhauer EA Wanders J Kaplan RS Rubinstein L Verweij J Van Glabbeke M van Oosterom AT Christian MC Gwyther SG New guidelines to evaluate the response to treatment in solid tumors. European Organization for Research and Treatment of Cancer, National Cancer Institute of the United States, National Cancer Institute of Canada J Natl Cancer Inst 2000 92 205 216 10.1093/jnci/92.3.205 10655437

[B9] Rice TW Rusch VW Ishwaran H Blackstone EH Worldwide Esophageal Cancer Collaboration. Cancer of the esophagus and esophagogastric junction: data-driven staging for the seventh edition of the American Joint Committee on Cancer/International Union Against Cancer Cancer Staging Manuals Cancer 2010 116 3763 3773 10.1002/cncr.25146 20564099

[B10] Uno T Isobe K Kawakami H Ueno N Kobayashi H Shimada H Mastubara H Okazumi S Nabeya Y Shiratori T Ochiai T Kawata T Ito H Efficacy and toxicities of concurrent chemoradiation for elderly patients with esophageal cancer Anticancer Res 2004 24 2483 2486 15330202

[B11] Onozawa M Nihei K Ishikura S Minashi K Yano T Muto M Ohtsu A Ogino T Elective nodal irradiation (ENI) in definitive chemoradiotherapy (CRT) for squamous cell carcinoma of the thoracic esophagus Radiother Oncol 2009 92 266 269 10.1016/j.radonc.2008.09.025 18952308

[B12] Yano M Takachi K Doki Y Miyashiro I Kishi K Noura S Eguchi H Yamada T Ohue M Ohigashi H Sasaki Y Ishikawa O Matsunaga T Imaoka S Prognosis of patients who develop cervical lymph node recurrence following curative resection for thoracic esophageal cancer Dis Esophagus 2006 19 73 77 10.1111/j.1442-2050.2006.00543.x 16643173

[B13] Watanabe M Nishida K Kimura Y Miyazaki M Baba H Salvage lymphadenectomy for cervical lymph node recurrence after esophagectomy for squamous cell carcinoma of the thoracic esophagus Dis Esophagus 2012 25 62 66 10.1111/j.1442-2050.2011.01215.x 21676066

[B14] Kersting S Konopke R Dittert D Distler M Rückert F Gastmeier J Baretton GB Saeger HD Who profits from neoadjuvant radiochemotherapy for locally advanced esophageal carcinoma? J Gastroenterol Hepatol 2009 24 886 895 10.1111/j.1440-1746.2008.05732.x 19655439 PMC4182869

[B15] Stahl M Wilke H Stuschke M Walz MK Fink U Molls M Siewert JR Schroeder M Makoski HB Schmidt U Seeber S Vanhoefer U Clinical response to induction chemotherapy predicts local control and long-term survival in multimodal treatment of patients with locally advanced esophageal cancer J Cancer Res Clin Oncol 2005 131 67 72 10.1007/s00432-004-0604-5 15480782 PMC12161171

[B16] Zhong Z Gu X Zhang Z Wang D Qing Y Li M Dai N Recombinant human endostatin combined with definitive chemoradiotherapy as primary treatment for patients with unresectable but without systemic metastatic squamous cell carcinoma of the oesophagus Br J Radiol 2012 85 1019 e1104 e1109 10.1259/bjr/15321801 22898155 PMC3500809

[B17] Piessen G Briez N Triboulet JP Mariette C Patients with locally advanced esophageal carcinoma nonresponder to radiochemotherapy: who will benefit from surgery? Ann Surg Oncol 2007 14 2036 2044 10.1245/s10434-007-9405-9 17453293

[B18] Murakami M Kuroda Y Matsusue S Okamoto Y Nakajima T Nishimura S Kusumi F Hajiro K Takeda H Treatment results of esophageal carcinoma of clinical T3, T4M0: historical comparison between neoadjuvant chemoradiotherapy followed by surgery or definitive radiotherapy and conventional surgery Oncol Rep 2000 7 571 578 10767370 10.3892/or.7.3.571

[B19] Watanabe M Nagai Y Kinoshita K Saito S Kurashige J Karashima R Hirashima K Sato N Imamura Y Hiyoshi Y Baba Y Iwagami S Miyamoto Y Iwatsuki M Hayashi N Baba H Induction chemotherapy with docetaxel/cisplatin/5-fluorouracil for patients with node-positive esophageal cancer Digestion 2011 83 146 152 10.1159/000321797 21266808

[B20] de Langen AJ Raijmakers P Riphagen I Paul MA Hoekstra OS The size of mediastinal lymph nodes and its relation with metastatic involvement: a meta-analysis Eur J Cardiothorac Surg 2006 29 26 29 10.1016/j.ejcts.2005.10.002 16337397

